# Oral mucosal lesions during SARS-CoV-2 infection: a case series and literature review

**DOI:** 10.1186/s43163-022-00203-3

**Published:** 2022-02-04

**Authors:** Mohammad Salah Mahmoud, Mohamed Shehata Taha, Ossama Ibrahim Mansour, Eman Barakat, Samar Abd Allah, Azza Omran, Anas Askoura

**Affiliations:** 1 Otorhinolaryngology Department, Faculty of Medicine, Ain Shams University, Cairo, Egypt; 2 Tropical Medicine Department, Faculty of Medicine, Ain Shams University, Cairo, Egypt; 3 Dermatology and Venereology Department, Faculty of Medicine Ain Shams University, Cairo, Egypt; 4 Clinical Pathology Department, Al-Mataria Teaching Hospital, Cairo, Egypt

**Keywords:** Oral lesions, Mucosal lesion, COVID-19, Ulcer, Sars-CoV-2

## Abstract

**Background:**

The most common manifestations of infection with COVID-19 are fever, sore throat, dry cough, headache, and body aches. The available evidence for successful and safe pharmacological therapy against COVID-19 has not yet been identified, and the possible evidence relates to many adverse reactions. Taste disorders, petechiae, desquamative gingivitis, unspecific oral ulcerations, xerostomia, and candidiasis are the oral manifestations related to SARS-CoV-2 infection.

**Main body of the abstract:**

We reviewed the literature regarding the reported oral mucosal lesions in cases with confirmed COVID-19 infection together with presenting five cases with oral mucosal lesions associated with COVID-19 infection. Direct causal association between COVID-19 infection and oral mucosal lesions is still vague, hence further research is required.

**Conclusion:**

Oral examination is mandatory in cases with suspected or confirmed COVID-19 infection.

## Background

Since the COVID-19 pandemic in December 2019, more than 148,343,515 individuals have been affected by COVID-19 [1]. Fever, sore throat, dry cough, body aches, headache, and rhinorrhea are the most common symptoms followed by dyspnea, anosmia, dysgeusia, and diarrhea [2].

Most human COVID-19 cases are mild (80%), while 20% may progress to severe disease and 5% may progress to acute respiratory distress syndrome, requiring intensive care unit admission [3].

Recent researches showed that human cells are invaded by the coronavirus via the receptor angiotensin-converting enzyme 2 (ACE2) [4]. Thus, cells which have ACE2 receptors may become host cells for the virus and cause an inflammatory response in related tissues, such as the salivary gland and mucosa of the tongue [5]. Interaction of SARS-CoV-2 and ACE2 receptors can also weaken sensitivity to taste buds, that may cause gustatory dysfunction [6].

The available evidence for successful treatment against COVID-19 has not so far been recognized, and the potential cures relate to many side effects [7]. Acute COVID-19 infection, with associated treatments, may also lead to adverse oral health outcomes. The oral manifestations related to COVID-19 are taste alterations, petechiae, gingivitis, vague ulcerations, xerostomia, and candidiasis [8, 9].

Recently, SARS-CoV-2 has been found in the saliva of infected patients. Additionally, ACE2 has been detected in oral mucosa, especially with more concentration on the tongue dorsum and salivary glands compared to the mucosa of the buccal cavity or the palates [10].

While some common oral signs or symptoms may contribute to early diagnosis of COVID-19 infection, no solid evidence is verifying whether these symptoms are the result of direct SARS-CoV-2 infection, a systemic response given the possibility of the impaired immune system, coinfections, or side effects related to treatment [11].

## Cases description

### Case1

**Fig. 1:**
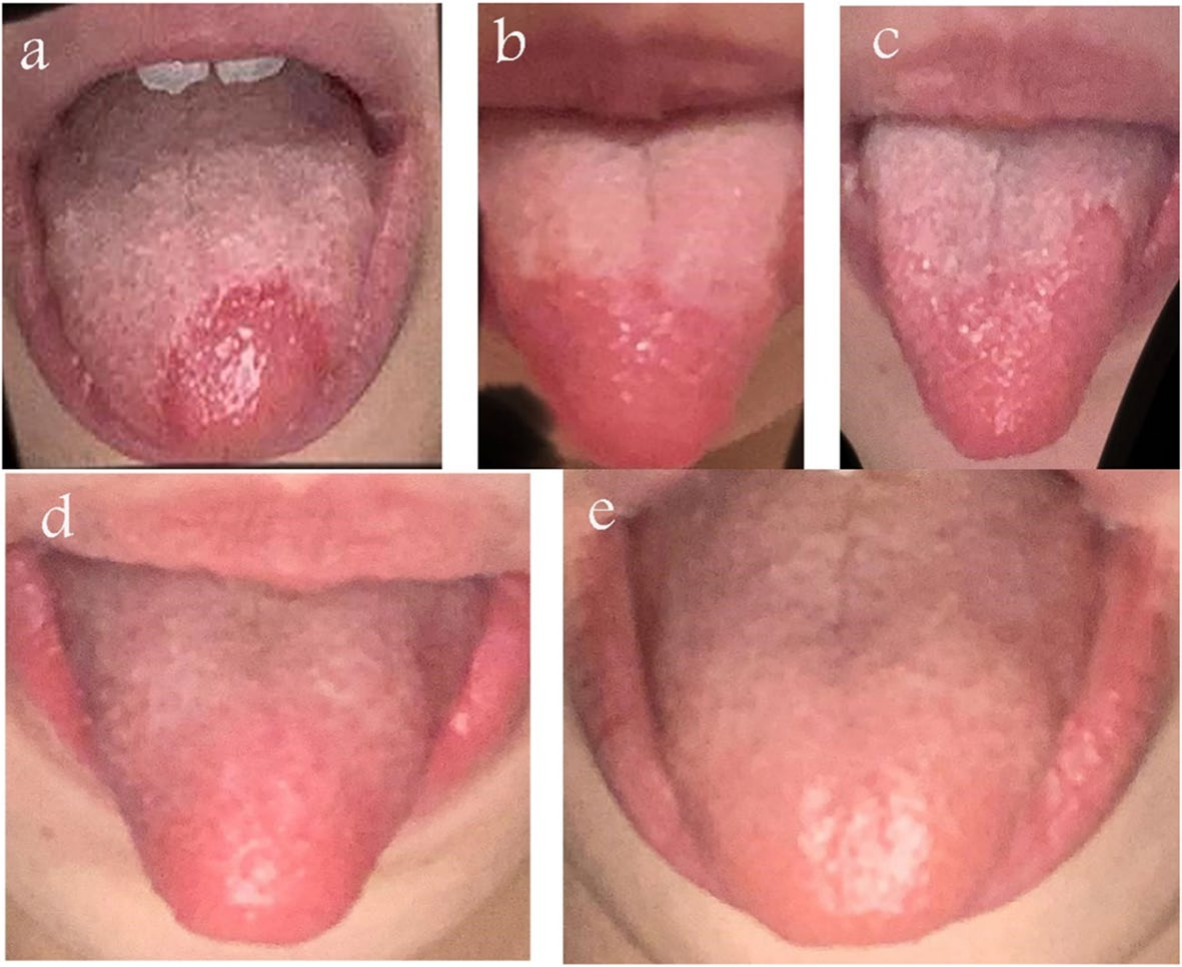
Clinical appearance of the tongue from day 3 (**a**) to day 10 (**e**)

On 23 September 2020, an 18-year-old Egyptian female presented with low-grade fever, malaise, and dry cough. She had no history of any chronic medical disorder and was not on any medications. Polymerase chain reaction (PCR) for SARS-CoV-2 was positive. Chest computed tomography (CT) was normal. All laboratory investigations, including complete blood picture (CBC), D. dimer, serum ferritin, and lactic dehydrogenase (LDH) were normal; only C-reactive protein (CRP) was positive. The patient started home isolation with medical treatment in the form of (Paracetamol 500 mg tablet three times daily, Azithromycin 250 mg capsule two capsules once daily for 5 days, vitamin C 1 gm once daily, Zinc 50 mg Tablet once daily, Lactoferrin sachets once daily and dextromethorphan syrup as cough suppressant 15 ml three times daily). On day 3, she developed a painless and non-itchy smooth glossy area that started on the tongue’s tip (Fig. 1a) and then extended to involve its dorsal anterior one-third (Fig. 1b, c). The surface appeared somewhat depapillated with a well-defined border.

Glossitis as a possible manifestation of COVID-19, a reaction from drugs, or candidiasis was considered. For this, local therapy in the form of a gel, containing Lidocaine hydrochloride and Cetylpyridinium chloride three times daily was added. By the 5th day, she complained of loss of smell and taste; therefore (Mometasone furoate monohydrate ) nasal spray two puffs in each nostril daily, saline 0.9% nasal irrigation three times daily, and smell training were added to her medications. Three days later, the sense of smell and taste returned near to normal. Most of the presenting symptoms were relieved by day 10 and the tongue lesion started to fade up (Fig. 1d, e).

### Case 2

**Fig. 2:**
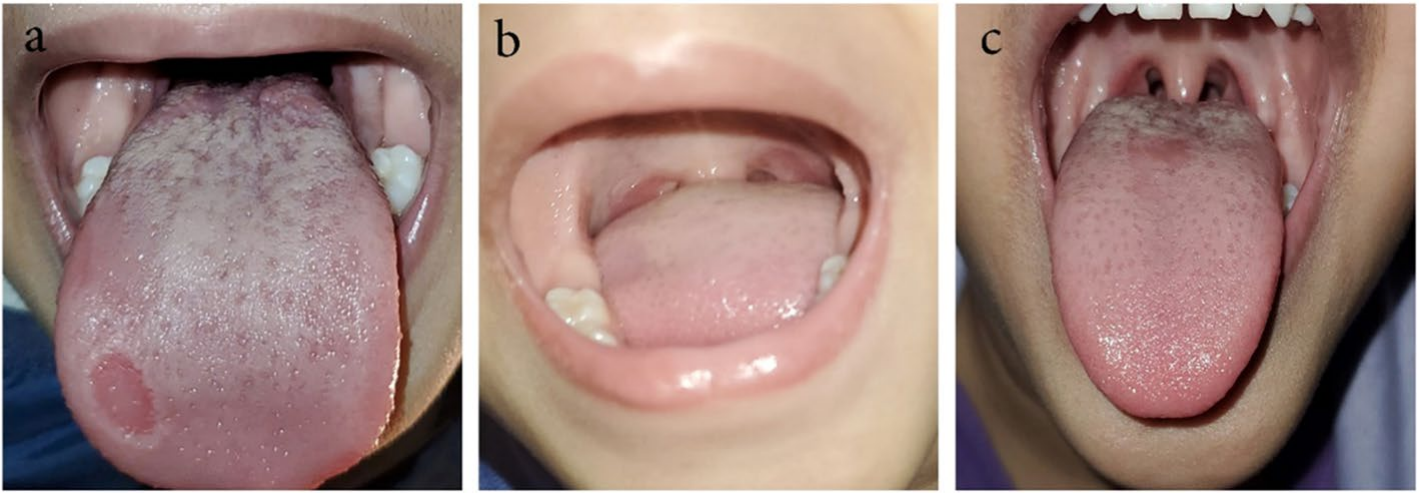
Clinical appearance of the tongue from day 5 (**a**), day 9 (**b**) to day 13 (**c**)

A 3-year-old girl complained of dry cough for 3 days, then fever developed (41 °C), associated with malaise. Full labs were done and CRP 156, relative lymphopenia, and normochromic normocytic anemia. She had no associated comorbidities and was not taking any medications. Polymerase chain reaction (PCR) for COVID-19 was positive. Chest X-ray showed right regular para hilar lower lung zonal soft tissue density showing air bronchogram inside, silhouetting the right cardiac border denoting right middle lobar abnormality. Two days later, she developed single erythematous erosion (depapillation) on the side of the anterior third of the tongue associated with a white coat mostly on the posterior two thirds with prominent tongue papillae in some areas (Fig. 2a). She received amoxicillin-clavulanic (90 mg/kg) together with acetaminophen, ibuprofen, and bronchodilator. By the 9th day, the oral lesion began to fade (Fig. 2b) then disappeared on day 13th (Fig. 2c).

### Case 3

**Fig. 3:**
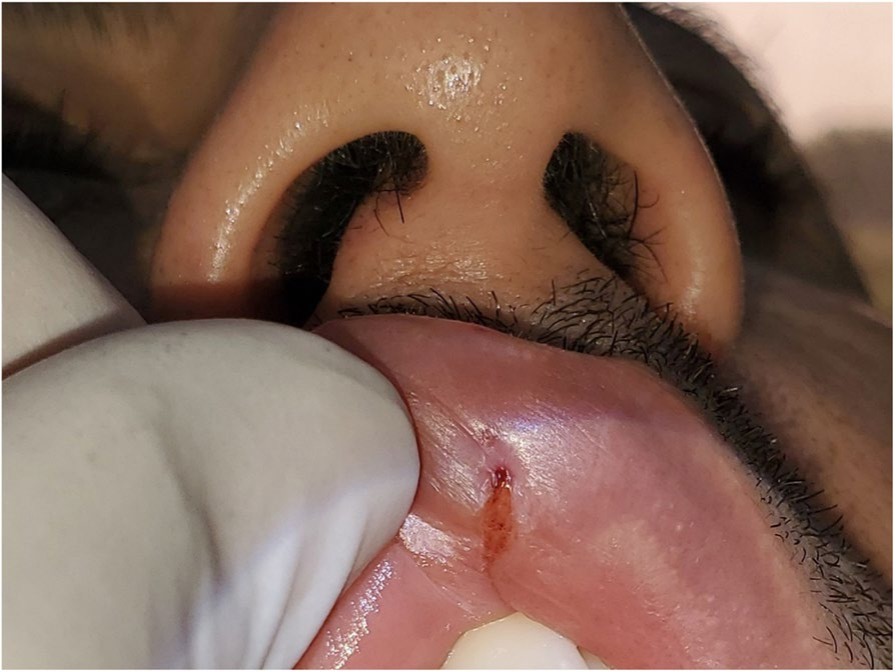
Clinical appearance of the lip

A 32-year-old male complained of sore throat, myalgia, headache, and rhinorrhea. Three days later, total anosmia and ageusia developed. COVID-19 infection was documented by a nasopharyngeal swab PCR. All laboratory investigations, including complete blood picture (CBC), D-dimer, serum ferritin, CRP were normal. On the fifth day, a painful cracked upper lip developed (Fig. 3). He received medications in the form of acetaminophen 500 mg thrice daily, Azithromycin 500 mg once daily for 6 days, vitamin C 1 gm once daily, Zinc 50 mg once daily, and local anesthetic for the oral lesion. the condition improved on the 8th day. The oral lesion disappeared on the 10th day.

### Case 4

**Fig. 4:**
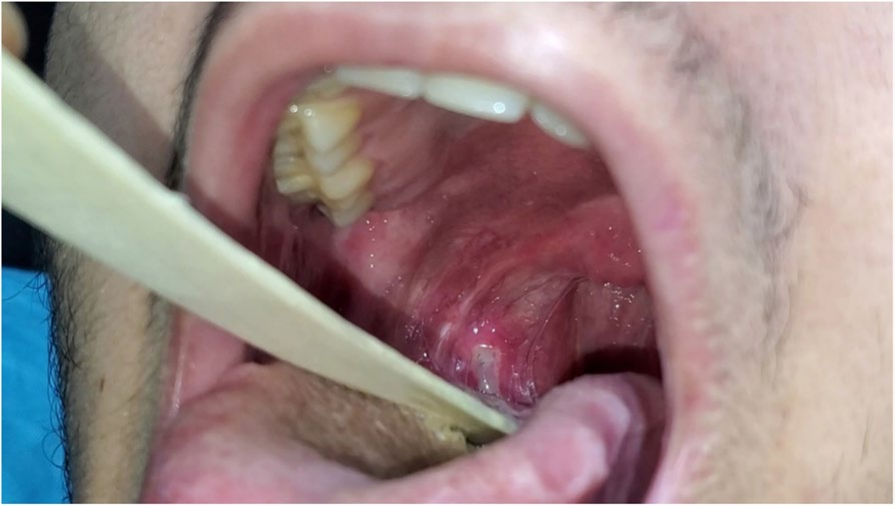
An ulcer on the right lateral oropharyngeal wall

A 19-year-old-male had a close contact with a positive COVID-19 family member, 4 days later, he started to complain of myalgia, headache, sore throat. Mouth examination revealed a painful ulcer on the right side of lateral pharyngeal wall ( he had a history of tonsillectomy, while no history of recurrent aphthous ulceration ) with irregular margins (Fig. 4). All laboratory investigations were normal except CRP was 90. A positive PCR for COVID-19 infection was documented. He received Azithromycin 500 mg for 3 days, antiseptic mouthwash (chlorhexidine), acetaminophen 1 gm twice daily. the condition improved on the 5th day. The oral ulcer disappeared on the 12th day.

### Case 5

**Fig. 5:**
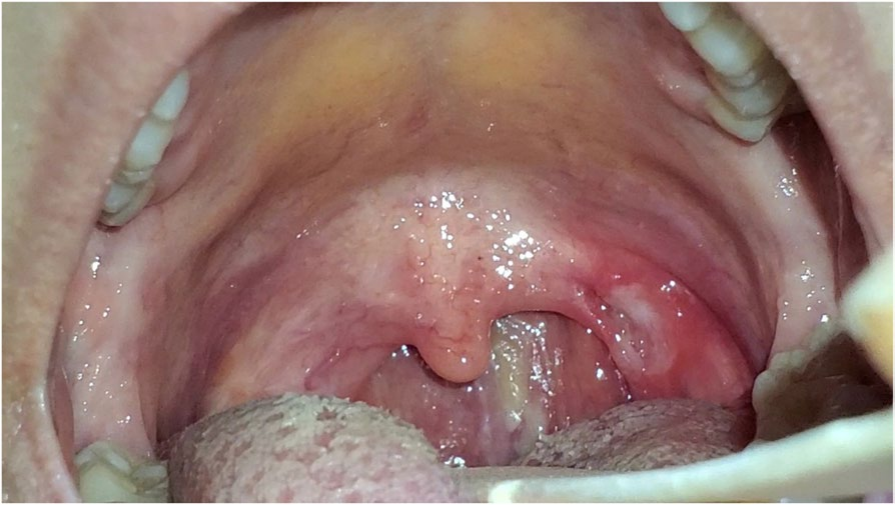
An ulcer on the left lateral oropharyngeal wall

A 23-year-old female complained of fever (38.5 °C), sore throat, and rhinorrhea; 3 days later, she had partial anosmia and ageusia, and nasopharyngeal swab was done for PCR which came back positive for Sars-CoV-2. She received azithromycin 500 mg daily for 3 days, acetaminophen 500 mg three times daily, vitamin C, and zinc supplements. Two days later, her sore throat became worse and localized to the left side. She sought medical advice. On examination, she had an ulcer (Fig. 5) on the left lateral oropharyngeal wall (she had a history of tonsillectomy and history of recurrent aphthous ulceration), with irregular margin and surrounding erythema. The ulcer disappeared after 12 days.

## Discussion

Despite the outline of several manifestations of SARS-CoV 2 [12] in the literature, some manifestations are still not described or not entirely confirmed.

The main ways of spread are direct, as in coughing, sneezing, and micro-drops of saliva during talking, or indirect, by interaction with mucous membranes in the body such as those of oropharyngeal, sinonasal, and ocular mucosae [13, 14].

We describe oral lesions in the form of two asymptomatic erythematous depapillated areas on the tongue’s anterior third, another lesion in the form of a painful cracked upper lip, and an ulcer on the lateral oropharyngeal wall. Whether it is a direct manifestation of COVID-19 or a drug reaction, or secondary fungal infection could not for sure be clarified. However, drug reaction is a less likely possibility as the condition improved without stopping any drugs.

Several types of oral lesions were described to date in confirmed COVID-19 patients. The commonest locations of oropharyngeal lesions were tongue, labial mucosa, palate, gingiva, buccal mucosa, oropharynx, and tonsil; (38%), (26%), (22%), (8%), (5%), (4%), (1%) respectively. Sixty-eight percent of the lesions were symptomatic (painful, or pruritic). Both genders were nearly equal in incidence of the oral mucosal lesions and healing time were in-between 3 and 28 days after appearance [15].

Carreras-Presas et al. described three cases with oral mucosal vesiculo-bullous lesions in SARS-CoV 2 positive [9]. The lesions were in the form of blisters or painful ulcers. All were cured and disappeared in 3–10 days. The authors claimed that the pain may be due to the higher expression of ACE2 in the surface mucosal cells of the tongue, compared to the gingival or buccal mucosa [16].

Brandao et al. reported eight cases of SARS-CoV 2 infection, with aphthous ulcerations affecting the tongue, lips, palate, and oropharynx. They detected that the evolution of the oral lesions and the healing were matching the resolution of the COVID-19 infection [6].

Malih et al. reported a 38-year-old patient who had laryngeal inflammation with an aphthous ulcer on the left tonsil [17]. Kämmerer et al. reported a 46-year-old patient with several grey membrane covered and well-circumscribed ulcers on the oral cavity.

They found increased levels of interleukin 6, positive herpes simplex virus (HSV) antibodies (IgM), and positive HSV PCR; hence, they proposed it was due to herpetic stomatitis [18]. While Dominguez-Santas et al. reported four cases with minor aphthous ulcerations on lips, buccal mucosa, tongue, and oropharynx. They found a negative HSV PCR together with negative serum for syphilis, HIV, EBV, CMV, HBV, and HCV. So they attributed the ulcers due to cytokine storm due COVID-19 infection [19].

Rodríguez et al. reported three cases with aphthous ulcers, burning, depapillation of tongue mucosa, and angular cheilitis [20]. Also, Tomo et al. reported a case of a 37-year-old female with painful erythematous depapillation of the lateral tongue border which healed within two weeks. They explained it by mucositis due to hypersensitivity to COVID-19 [21].

Also, Chaux-Bodard et al. [22] published a case of a tongue ulcer that occurred in a 45-year-old female patient. She complained for 1 day of a painful lesion of the tongue mucosa, followed by red macula, which progressed into irregular-shaped and asymptomatic ulcer. Ten days later, the ulcer fully cured without a scar.

Glavina et al. published a case of oral mucosal lesion in a 40-year-old patient with hard palatal recurring HSV, and the ventral surface of the tongue had non-specific white lesions with aguesia. The patient was given systemic antiviral therapy (Acyclovir) and local treatment (local antifugal; nystatin, and local anesthetic) for 14 days. The patient was completely cured after 21 days. In their opinion, the acute ageusia is due to SARS-CoV-2. While recurring HSV of the hard palatal mucosa were triggered by the stress induced by SARS-CoV-2 and so is likely to be a secondary symptom of the host’s weak immune system [23].

Tapia et al. reported four patients presented with hemorrhagic lesions, and stomatitis. They proposed the lesions were due to thrombi formation and vasculitis owing to COVID-19 disease [24].

Tomo et al. described a case of a 37-year-old woman, with fever, taste alteration, and anosmia. COVID-19 infection was confirmed by PCR. There were depapillation of the tongue borders together with diffuse erythema, they prescribed chlorhexidine 0.12% mouthwashes. The patient was asymptomatic after 2 weeks [21].

Ciccarese et al. reported a 19-year-old woman complained of fever, sore throat, altered smell, and asymptomatic skin and oral lesions. Physical examination revealed multiple ulcers, blood clots on the lips, and petechiae on the palate and gingiva. Ten days later, skin and mucosal lesions disappeared [25].

Ansari et al. described two cases, a 56-year-old woman complained of fever and dyspnea. After 5 days, mouth ulcers developed. on examination; several ulcers were found in the oral cavity (hard palate) with different sizes and irregular margins. The second case was a 75 years old male who complained of dysphasia. They found multiple red painful ulcers on the oral tongue. They took a biopsy from the lesions from both patients which revealed diffuses edema, mucosal erosions, and submucosal granulations [26].

Soares et al. described a case of a 42-year-old patient presented with fever, cough, chest tightness, cutaneous petechiae, and burning ulcers in the buccal mucosa. Several erythematous macules of varying sizes on the hard palate, tongue, and lips were found by examination. Oral mucosal lesions were disappeared after three weeks. Biopsies from the ulcers demonstrated an submucosal infiltration with chronic inflammatory cells along with necrotic areas and thrombi occluding the blood vessels. Negative Immunohistochemical reactions for common viruses that cause ulcers: HHV-1, HHV-2, CMV, treponema pallidum, and EBV. They consider the mucosal lesions might be associated with COVID-19 thrombotic vasculopathy [27].

Jimenez-Cauhe et al. published three cases complained of macules and petechiae on the palatal mucosa together with skin target lesions. Systemic steroid were prescribed with full recovery within 2–3 weeks [28].

Amorim et al. in their study concluded that there is no clear evidence of etiopathological association between oral mucosal lesions and SARS-CoV 2. The clinical associations indicate that impaired immunity, drugs side effects, and coinfections, rather than an oral mucosal primary etiology caused by SARS-CoV-2 [29],

The oral cavity is a point of entry for variety of microorganisms, including SARS-CoV-2, which could be discovered in COVID-19 infected patients’ saliva, proposing that virus shedding in the saliva is linked to disease symptoms [30].

These oral mucosal lesions, develop concomitant with hyposmia/ageusia, or up to 14 days later. Interestingly, healing of these mucosal lesions occurs corresponding with the resolve of SARS-CoV 2 infection which might represent an association between viral infection, oral lesions, and their disappearance [31].

The manifestations of COVID-19 have been linked to etiological factors involving a “cytokine storm” caused by an excess production of proinflammatory cytokines as a result of a dysfunctional immune reaction [32]. Furthermore, it has been suggested that proinflammatory cytokines from inflamed gingiva, such as IL-1β and TNF-α may infiltrate saliva [32, 33].

Although causal association between SARS-Cov 2 infection and the oral mucosal lesions cannot be recognized. There are plenty of variables that could trigger oral mucosa lesions such as stress caused by social life limitations during the lockdown, oral hygiene issues [34], herpes simplex virus, glossitis, oral candidiasis, drug-induced reactions, autoimmune disorders, and nutritional deficiency [16].

## Conclusion

Clinical oral inspection may help to conduct a better initial triage and identify likely early manifestations of the SARS-CoV-2 infection, and it should be a standard part of the protocol of examination in all patients with confirmed SARS-CoV-2 infection. Thorough research is needed to explain the association between oral mucosal lesions and COVID-19 and to declare the previously exposed theory.

## Data Availability

Available upon request.

## Abbreviations

ACE2: Angiotensin-converting enzyme 2
PCR: Polymerase chain reaction
CT: Computed tomography
CBC: Complete blood picture
LDH: Lactic dehydrogenase
CRP: C-reactive protein
HSV: Herpes simplex virus
HIV: Human immunodeficiency virus
EBV: Epstein-Barr virus
CMV: Cytomegalovirus
HBV: Hepatitis B virus
HCV: Hepatitis C virus
HHV: Human, herpesvirus
IL: Interleukin
TNF: Tumor necrosis factor
